# A Spatial Decision-Support Framework for Assessing Road Network Efficiency in Emergency Healthcare Accessibility: A Case Study of Eastern Province Cities, Saudi Arabia

**DOI:** 10.3390/healthcare14213734

**Published:** 2026-11-03

**Authors:** Sultan Alamri

**Affiliations:** College of Computing and Informatics, Saudi Electronic University, Riyadh 11673, Saudi Arabia; salamri@seu.edu.sa

**Keywords:** GIS, road network analysis, accessibility analysis, emergency medical services, smart cities, health services

## Abstract

Background: The pressure on emergency medical services (EMSs) has been exacerbated by increased urbanization and population growth in major Saudi cities such as Dammam, Al Khobar, and Dhahran. In this study, a spatial decision-support framework is proposed to determine the accessibility to ambulance services and hospitals. Methods: Within the spatial decision-support framework, modeled two-leg travel time (station-to-scene plus scene-to-hospital, including modeled traffic-signal delays) is evaluated under both peak and non-peak traffic conditions, using a 30 min travel-time threshold. The model comprises road network data, ambulance and hospital locations, and traffic conditions to evaluate the travel component of emergency healthcare accessibility and to identify areas that are disadvantaged. Times for dispatch, ambulance response, scene assessment and treatment, stabilization, and loading are not included. Results: The results reveal significant spatial and temporal disparities, with accessibility decreasing during peak hours. Al Khobar has relatively better accessibility, while Dammam is consistently and significantly underserved, and Dhahran has moderate access. Taking into account the population data, it is evident that unequal accessibility is due to both spatial and demographic factors, with a significant number of residents having poor access to EMS. Conclusions: The findings can inform the improvement of EMS planning and people’s access to equitable healthcare. The framework can support the connection of underserved areas and direct development in the spatial distribution of emergency healthcare services.

## 1. Introduction

The term “healthcare services” comprises hospitals and ambulances. Healthcare is a basic human right essential for the overall well-being of society, and ready access to necessary medical services is a key element of protecting that right, particularly in cases of emergency [1,2]. In Saudi Arabia, ready access to prompt emergency medical services (EMSs) is becoming a crucial issue [3,4] due to increasing urbanization and population growth. It is essential to guarantee efficient and quick medical response times during emergencies as Saudi Arabia’s population and cities continue to grow [1,5].

Given the growth of urban areas and the subsequent problem of traffic congestion, the prompt delivery of emergency medical services has become extremely difficult, particularly in the case of medical emergencies where the choice between a prompt and a delayed response may mean the difference between life and death [6,7]. Quick access to emergency assistance is essential for saving lives and minimizing the severity of trauma. By determining the spatial accessibility to healthcare services, we can reduce response times and improve the general safety and well-being of the community—an important aspect of Saudi Vision 2030 [5,8,9]. People feel more secure knowing that they can receive prompt emergency medical services when required. Therefore, when strategically assigning emergency medical services, those areas with poor spatial accessibility must be given priority [10,11]. The key to ensuring the safety and health of the community is to identify such areas and strategically distribute resources accordingly. This approach will help to establish a more secure and resilient society where healthcare services are easily accessible when most required.

In this paper, we propose a new analytical model for evaluating medical emergencies by assessing spatial accessibility to EMSs using road networks and Geographic Information System (GIS) technologies. The study focuses on three major cities in the Eastern Province of Saudi Arabia: Dammam, Al Khobar, and Dhahran. The model determines the accessibility of ambulances and hospitals, based on modeled two-leg travel time. In this study, we specifically determine whether this travel time is within a 30 min threshold. This includes the modeled travel time from the ambulance station to the patient’s location, and then from the scene to the nearest major hospital with the necessary facilities for emergency care [12,13]. This threshold applies only to the travel times and does not indicate the total EMS response time, total prehospital time, or adherence with the “Golden Hour.”

The main objective of this study was to identify "black spots": inaccessible areas where, in the event of an emergency, the modeled two-leg travel time from the ambulance station to the patient’s location and then to the nearest emergency hospital exceeds 30 min. Moreover, the study compares subdistricts, cities, and administrative zones in terms of their levels of accessibility based on several factors, such as area size, population, and other demographic and geographic variables. The framework makes planning possible due to the identification and evaluation of gaps in accessibility; it is not intended for optimizing facility locations and ambulance deployment.

In this paper, Section 2 presents the background. The literature review is the focus of Section 3, together with a discussion of related work in the fields of spatial accessibility analysis, transportation networks, and emergency healthcare services. Section 4 describes the methodology: the data collection process, travel-time estimation, and the accessibility modeling framework. Section 5 presents the results and discussion, including both spatial and population-based analyses of accessibility. Section 6 concludes the paper, revisiting key findings and offering suggestions for future research directions.

## 2. Background

**Figure 1:**
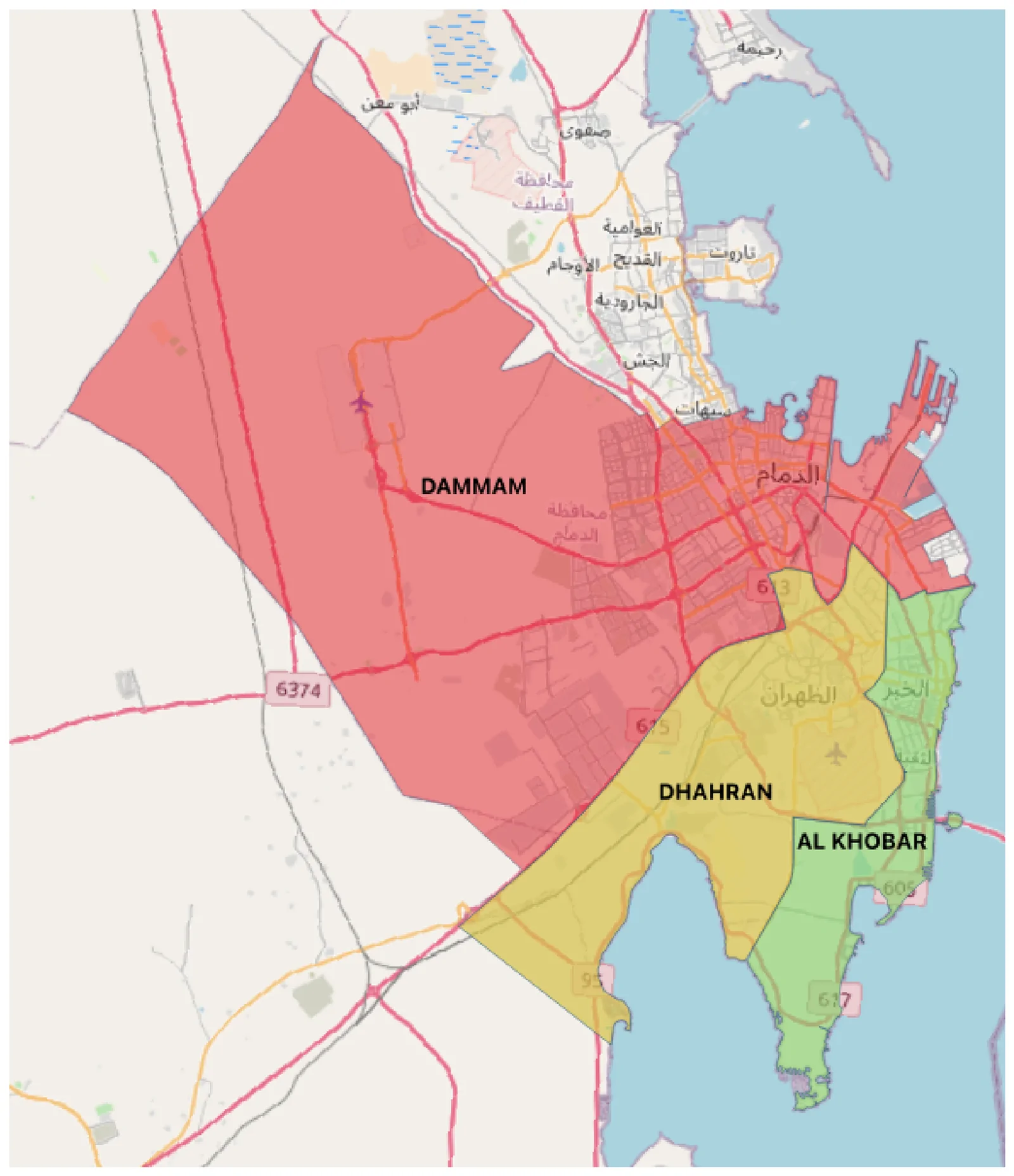
Dammam, Al Khobar, and Dhahran cities boundaries.

The focus of this study was the Dammam Metropolitan Area in the Eastern Province of Saudi Arabia, comprising the cities of Dammam, Al Khobar, and Dhahran. These adjacent cities constitute a contiguous urban region with a population totaling approximately 2.34 million residents, with approximately 1.5 million inhabitants in Dammam, around 700k in Al Khobar, and 143k in Dhahran as of 2022 [14]. Each city is a critical economic, residential, and healthcare hub within the region, hence the necessity of having efficient and reliable emergency healthcare services. Figure 1 illustrates the spatial arrangement of these three cities, indicating their geographic proximity and interconnectedness. Given the densely populated urban environment and the complex spatial features of the cities, it is essential to have a carefully planned medical emergency infrastructure that can effectively deliver adequate healthcare.

**Figure 2:**
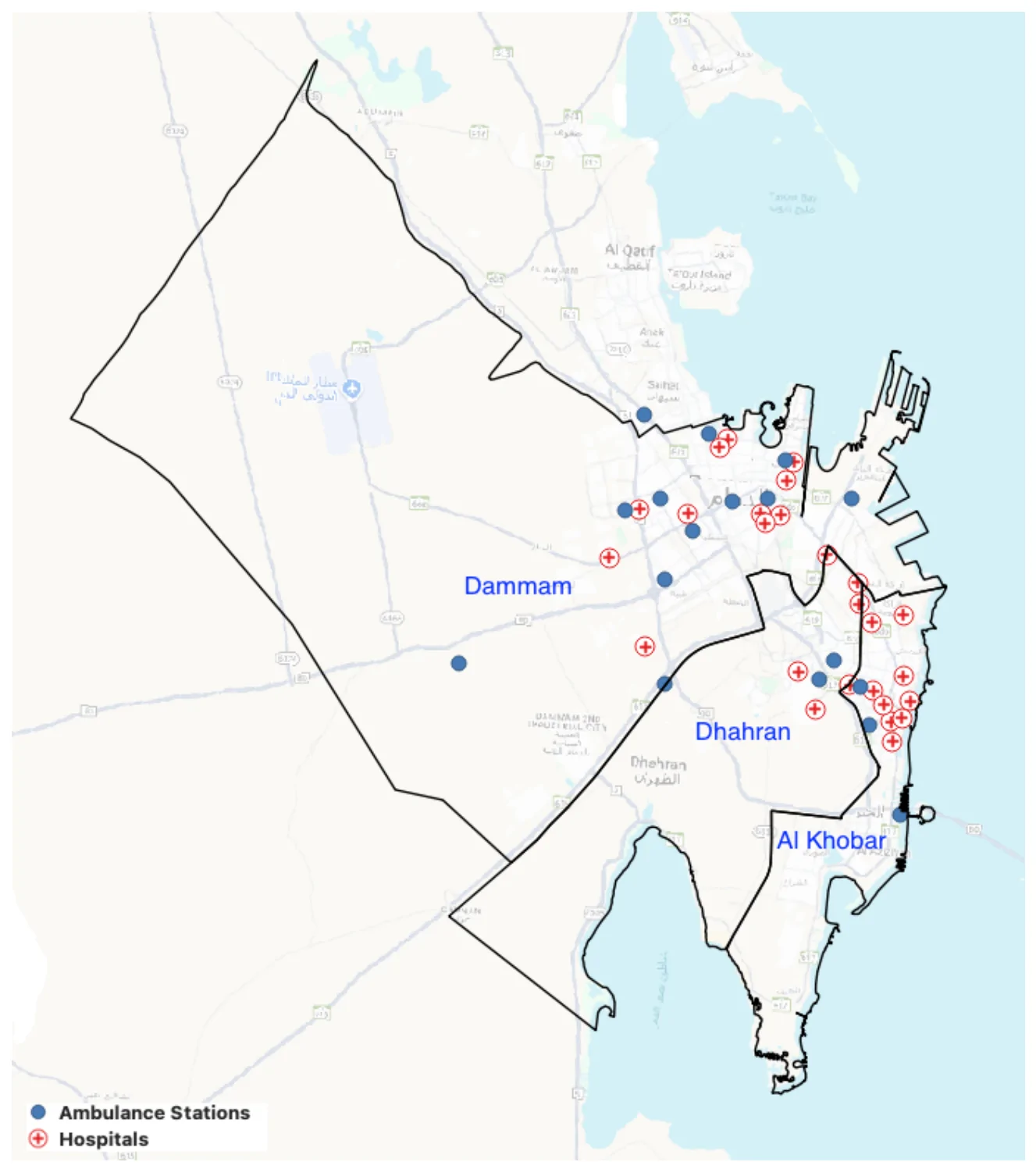
Dammam, Al Khobar, and Dhahran and the major hospitals and ambulance stations.

The current health infrastructure of these cities includes major hospitals equipped with fully operational emergency departments (EDs) and ambulance stations operated in the main by the Saudi Red Crescent Authority. Clinics and healthcare centers without comprehensive 24 h emergency services or advanced emergency care capabilities were excluded from this analysis. Figure 2 shows the locations of major hospitals and ambulance stations, indicating their positions relative to population centers and major transportation routes. Typically, the hospitals are located in densely populated areas and near major roadways. Although ambulance stations are strategically placed so as to reduce response times, certain peripheral areas are relatively far from these services.

An extensive amount of medical literature examines the importance of timely emergency care, emphasizing the concept known as the Golden Hour. This refers to the first hour after a severe injury has been sustained, while the connection between pre-hospital time and patient outcome is still uncertain [15,16]. Healthcare accessibility research emphasizes the significance of locating areas that in need of additional attention and development health care equality [11]. Spatial disparities in access to primary health care in urban neighborhoods of Canada were discovered by this study; poor areas are concentrated in urban fringes and around downtowns. Recent studies within Saudi Arabia have also raised similar concerns, emphasizing the importance of improving healthcare equity and outcomes by means of adhering to a 30 min standard [17].

## 3. Literature Review

Various GIS-based solutions have been developed to resolve complex issues associated with many areas of health and well-being. Spatial decision-support systems are likely to be a comprehensive solution ensuring better service delivery. The roles of those employed in the health sector are crucial as they are either directly or indirectly related to human health. The identification of catchment areas using all possible data sources, together with decision-support systems developed with the help of GIS technology, offers a number of benefits to society in general [1].

Ref. [18] developed a solution for emergency ambulance deployment by using spatial techniques to identify catchment areas and determine the best routes for emergency responses. The work uses the “Voronoi spatial model and time-based Dijkstra’s routing algorithm”. This technique applies the shortest travel-time approach instead of the shortest distance model. This model also involves an analysis of GIS-based spatial road network and accessibility. The area chosen for this study is the metropolitan region of Riyadh, in the KSA. The research helped to determine the ambulance station closest to the reported emergency within the catchment area.

Travel time is the most important factor when responding to an emergency in a particular location. Melbourne (Australia), a densely populated metropolitan city, was the focus of the research conducted by [19]. Travel time depends not only on the distance covered, but also on the condition of roads. To address these factors, the study used a travel-time-based accessibility analysis in union with Voronoi-based catchment model building. The catchment areas of each hospital were determined and the travel time to each were calculated. To compute the most realistic speed limits of the catchment zones, speed reductions, different waiting times, and all possibilities were taken into account by the researchers. By incorporating all these factors, the Travel Time Voronoi Diagram method was able to predict more reality-based estimations of routing.

By using geo-mapping to create a plan for future maternity care, ref. [20] set out to determine the number of women with uncomplicated pregnancies who are eligible for home deliveries in Victoria, Australia, and living within 15–25 km of appropriate maternity hospitals. It is estimated that 126,830 low-risk women gave birth in Victoria between 2015–2017, half of whom were within 25 km of seven Victorian hospitals. At the moment, the current public homebirth model applies to only 2% of eligible women. Based on the geospatial data, a random set of addresses was generated corresponding to the number of registered births per postcode. Minimal path algorithms were applied to Victoria’s Open Street Map (OSM) to calculate travel distances between addresses and public hospitals. Each hospital’s catchment area was determined by ascertaining the addresses closest to the hospital.

Public transport is the backbone of a transport system, and helps to decrease traffic congestion and air pollution. The research conducted by [21,22] focused on the issue of public transport inadequacies (Melbourne, Australia) to highlight the neglected parts of the area of interest. Catchment areas were derived spatially and blank spots were identified. These were those areas that lack public transport facilities, accounting for 5.09% of the residential category. Maps for the availability and frequency of travel by public transport with respect to the area are also given for policymakers to regulate both availability and the frequency as needed by those areas. It was also observed that some areas with low population density had little public transport. The collective results accumulated by the GIS spatial analysis of each factor help decision makers to improve the delivery of services to the general public [22].

Health emergency responses correlate with the technology and the infrastructure available in a certain vicinity. Ref. [23] proposed a hospital suggestion model for the NYC that involved compiling a dataset of travel time from different locations in the city via the road network. This static data was used to determine the minimal emergency response time for ambulances to reach different hospital locations according to their injury code. An arc map was used to analyse the road network. By integrating telematics data from the city vehicles with linear collaboration of road network, a pattern N was developed which decreased travel time for emergency responses in this densely populated city. Ref. [24] also closely investigated healthcare management based on the ambulance allocation model in Florida, USA. The researcher proposed an optimal solution for ambulance utilization, decreasing the overcrowding of healthcare facilities. In this research, single objective and bi-objective game theory methodology were applied. The proposed approach reduced overcrowding and the wait time for treatment. Nevertheless, it is crucial to select the appropriate approach so as to safeguard the well-being of all patients. Ref. [25] conducted a research study in south Delhi, India, focusing on the optimization of ambulance locations using temporal data of accident sites in the study area. Tabular data was spatially plotted for geographic analysis. The researcher conducted GIS-based hotspot analysis and calculated the travel time to determine new locations for ambulances in order to cover the area in less time. More induction and locations for ambulances were also proposed to further reduce the emergency response time.

The main responsibility of an emergency response unit is to administer pre-hospital medical care to the patient. Ref. [26] conducted a study on emergency responses in the urban–rural area of Vestfold, Norway. The aim of this research was to ascertain ways to ensure the optimal distribution of ambulances among urban and rural base stations. The MEXCLP model was used in this study, and urban and rural vicinities are termed “high”- and “low”-demand areas for emergency cases. This model also suggests having backup units in urban areas where there is likely to be greater demand, and single-unit coverage in rural areas due to lower demand. The researcher collected spatiotemporal data of incidents for the period of 2013–2016 and locational data of base stations. A demand-point classification technique was applied to determine the areas of high demand. All datasets along with data pertaining to population and previous incidents were incorporated to identify the most concentrated areas where more ambulances are required and to identify those underserviced areas where an emergency service base was required.

There are many studies that have addressed the aspects of EMS accessibility; however, they use artificial intelligence and machine learning [27,28]. For example, ref. [27] focuses on the prediction of demand for short-term EMS using nine machine-learning algorithms. Another example, ref. [29], focuses on developing machine learning models for ambulance travel-time estimation, where it focuses on using real-world spatiotemporal data (Skåne, Sweden). It also measured the influences of various factors such as traffic, weather and others. Ref. [30] focuses on the planning ahead of emergency-responder repositioning. This approach uses hierarchical multi-agent reinforcement-learning, which predicts and makes operational decisions; it also considers geographical accessibility under current service provision (in Nashville and Seattle). These also provide other avenues for further development.

The research that is included in this study considers many different factors of EMS accessibility, including travel-time routing and catchment definition [18,19], hospital indication [23], and ambulance dispatch and site indication [24,25,26]. Our model does not target optimization of resource allocation; instead, it basically focuses on assessing existing service networks. The novelty of the approach consists in the evaluation of the travel time from stations to patient location and then from that location to hospitals, with the comparison of peak-hour and non-peak accessibility at different scales—the city level and the neighborhood level, and linking the neighborhood accessibility to the population distribution.

## 4. Methodology

### 4.1. Study Area and Scope

The study focused on the Eastern Province Municipality of Saudi Arabia, specifically, on the tri-city metropolitan area comprising Dammam, Dhahran, and Al Khobar. These three adjacent cities constitute a major urban cluster with a combined population of approximately 2.34 million residents [14]. Geographically, the area features a dense primary road network connecting the cities, surrounded by rapidly growing suburbs and peri-urban districts. The area chosen for analysis consists of each city’s urban center as well as the outlying neighborhoods, ensuring that both city centers and suburban areas are examined to determine their accessibility to emergency healthcare.

### 4.2. Data Collection and Sources

The methodology integrates multi-source spatial data with historical traffic information. The key datasets and their sources include:

- Hospital locations: coordinates of hospitals and major healthcare facilities were sourced from the Saudi Ministry of Health and cross-referenced with GIS records from the Eastern Province Municipality to ensure comprehensiveness.
- Ambulance stations: the locations of emergency service centers, including both urban and suburban coverage, were obtained from the Saudi Red Crescent Authority.
- Road network and travel times: the spatial structure of the road network was constructed using OpenStreetMap (OSM) data, which offers freely available, high-resolution geographic information. This network was processed and analyzed using QGIS for spatial alignment, cleaning, and base-layer visualization. For dynamic travel-time estimation, Google Maps API was used, as it provides historical traffic-informed travel times based on typical driving conditions at different times of day [31].

The analysis included 25 hospitals and 15 ambulance stations: 11 hospitals and 10 stations in Dammam, 4 hospitals and 2 stations in Dhahran, and 10 hospitals and 3 stations in Al Khobar. Their locations are shown in Figure 2. As noted previously, only large hospitals with fully operational emergency departments were included. Those without full emergency services or clinics, and those that were in construction, were not included. Also, bed availability and ER workload were not modeled in the hospital selection. The hospitals’ location data was collected from the Ministry of Health in the month of September 2025. Similarly, the  ambulance stations’ locations were collected from the Saudi Red Crescent Authority in the month of September 2025. This analysis represents the layout of the facility from these datasets. The hospital and ambulance-station locations were examined and verified using Google Maps and physical visits where feasible.

### 4.3. Travel Time Estimation

**Figure 3:**
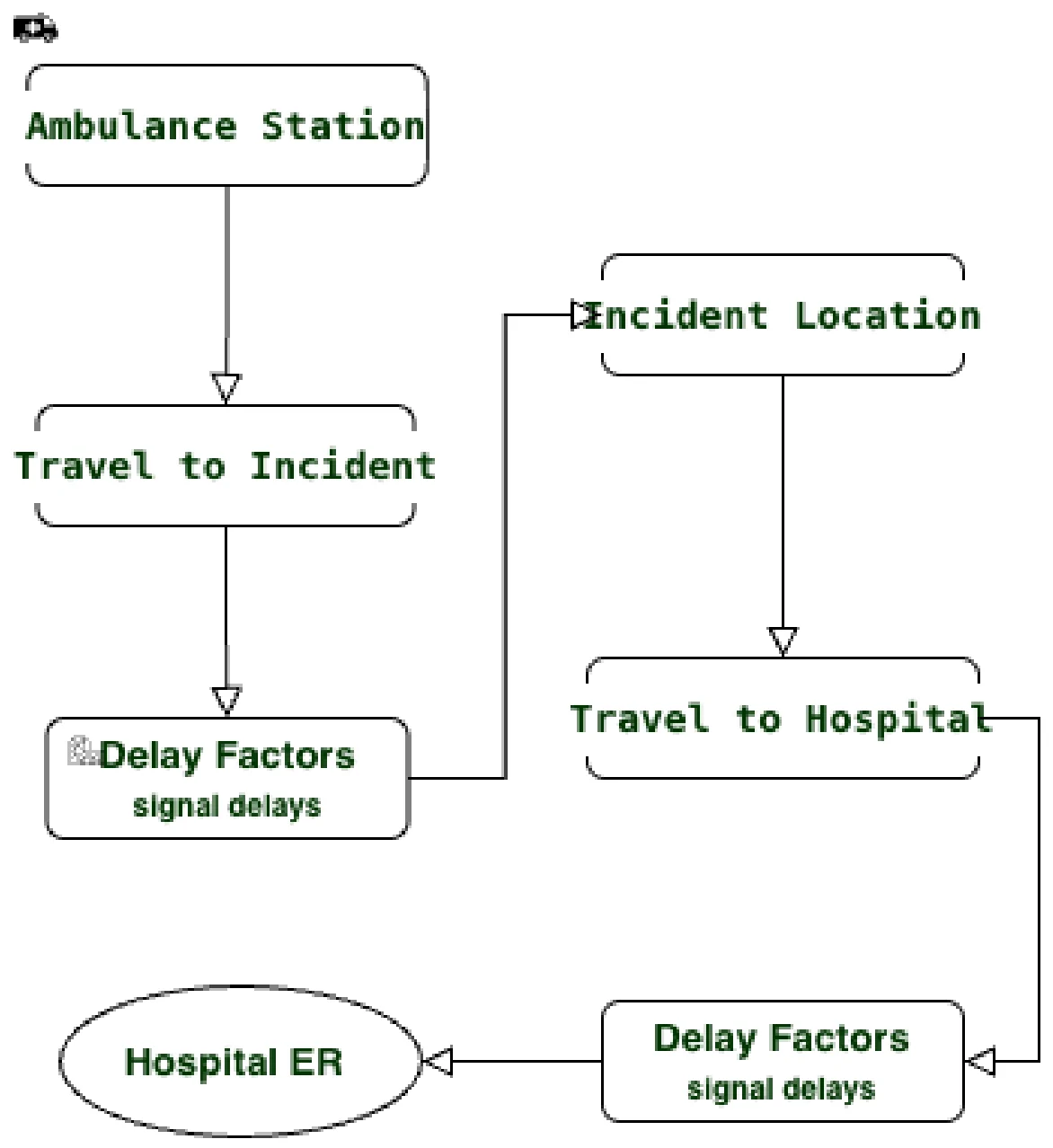
Modeled two-leg travel flow showing travel segments and delay factors (traffic-signal delays) between the station, incident location, and hospital ER.

Importantly, this study was conducted to estimate the modeled two-leg travel time of an ambulance traveling from its base station, to the patient, and then to the nearest hospital. The key performance metric is whether this modeled travel time is within the 30 min threshold. Figure 3 depicts the two travel legs and linked with the traffic-signal delays. The model considers only travel time and modeled traffic-signal delays; other EMS operating times are not modeled.

To account for the daily variability of traffic conditions, separate analyses were conducted for:

- Peak hours: weekday morning (7–9 a.m.) and evening (4–6 p.m.);
- Non-peak hours: midday and late evening.

For each of these time periods, estimated driving travel durations were obtained via Google’s API. Travel times were retrieved for each pair: ambulance station → incident location, and incident location → hospital (Figure 3). Travel durations were extracted from the duration_in_traffic field. This set of API durations became the base inputs prior to the independent traffic-signal timing discussed in Section 4.4.

Historical travel-time retrieval: a script interfacing with the Google Maps Distance was created to collect travel durations for these routes across the study area. The researchers used a dense grid of potential incident coordinates, and results were compiled into a large origin–destination matrix [1,32].

### 4.4. Incorporating Traffic-Signal Delay

The additional signal-delay correction factor was intended through repeated manual checks, which suggests results closer to the actual travel times on selected routes. It was *intended to consider potential day-to-day local delays* not fully considered by the baseline estimates, although some signal effects may have been already reflected in the API estimates. This extra adjustment was made as an extra supplementary for locally experienced travel-time under-estimation. These exploratory checks provided justification for the adjustment; the per-signal delay and stopping probability were assumed, not estimated from the signal-level measurements. A custom traffic-signal delay calculator was developed by means of the following logic:

- Route intersection data were parsed to identify signalized junctions along both travel legs.
- An average red-light delay (45 s) was assumed per signal, as well as a stopping probability of 0.5. This gives an anticipated additional delay of 22.5 s per signal. No random sampling was used, and the similar parameters were applied for both peak and non-peak cases. These were maintained for both time periods (peak and non-peak), representing a simple modification that was designed to improve the credibility of travel times, although delays may vary between periods depending of the actual intersection. Note that there was no traffic-priority modification for emergency vehicles.

The total accumulated travel time for a leg was calculated with the following:

$$Ti_{\mathrm{augmented}}=Ti_{\mathrm{API}}+Ti_{\mathrm{signal}\_\mathrm{delay}}$$

### 4.5. Accessibility Modeling

The accessibility assessment was carried out to determine whether the modeled two-leg travel time was within 30 min; this was done by summing the two segments of the trip and the delay. The threshold of 30 min was selected as an indicator of geographical accessibility based on the fact that it was used in other studies of accessibility to emergency care [12,13]. However, there are different elements of the journey included in previous studies, while in this research, the cut-off is applied to the travel time of two legs of travel. This resulted in two binary maps—one for peak, and one for non-peak conditions—indicating whether each location was accessible or constituted a black spot. Note that populations do not influence the binary classification; the populations of neighborhoods are involved in the further population analysis.

### 4.6. Comparative City-Level Analysis

Coverage was evaluated by comparing the percentage of study area in each city categorized as accessible within the 30 min modeled two-leg travel-time threshold. We also examined the average emergency travel times and the distribution of black spots:

- Districts with persistent black spots were identified.
- City-wide averages were calculated for Dammam, Dhahran, and Al Khobar.
- Traffic congestion was analyzed by observing and comparing the number of black spots in the peak and non-peak maps.

These comparisons aid in assessing which city has superior EMS accessibility, and where infrastructure or resource allocation improvements are needed. Algorithm 1 illustrates the emergency coverage and black spot determination process.
**Algorithm 1** Emergency Coverage and Black Spot Determination  1:**for** traffic_condition ∈ {Peak, nonPeak} **do**  2:      **for** incident_location *i* in StudyAreaGrid **do**  3:            status[i]←**Black Spot**                                                                      ▹ Default state  4:            nh←findNearestHospital(i)  5:            **for** ambulance_station *s* in AmbulanceStations **do**  6:                  $ti_{1}\leftarrow \mathrm{getTravelTime}(s,i,$traffic_condition)  7:                  $ti_{2}\leftarrow \mathrm{getTravelTime}(i,nh,$traffic_condition)  8:                  N1 ← number of signals along s → i  9:                  N2 ← number of signals along i → nh10:                  delay1 ← N1 × 0.5 × 45 / 6011:                  delay2 ← N2 × 0.5 × 45 / 6012:                  **if** ($ti_{1}+ti_{2}+delay1+delay2$) ≤ 30 min **then**13:                        status[i]← **Accessible**14:                        **break**                                                                    ▹ Accessibility confirmed15:                  **end if**16:            **end for**17:      **end for**18:**end for**

### 4.7. Exploratory Travel-Time Checks

The manual check exercise was performed manually during experiments in selected locations in Dammam, Dhahran, and Al Khobar. Purposeful sampling was done to focus on neighborhoods located within and border the 30 min threshold from the accessible category. These were done across the three cities by adopting purposive selection rather than a fixed sampling scheme. Locations that are close to hospitals did not attract much focus due to the higher chances of being accessible in those locations. The verification exercise involved actual travel when possible and comparisons with the google map estimates of current times (for both peak and non-peak conditions). The validations included physical journeys where feasible, along with comparisons against google maps, which served as consistent validations instead of independent ground truth.

In these selected case validations, the additional signal-delay adjustment helped to develop agreement with noticed travel times or current google map rates. These validations/checks were qualitative in nature: it considered both peak and non-peak times. The aim was to assess, improve and evaluate the plausibility of the estimates; however, quantitative error statistics were not calculated. These validation results were therefore supportive of a plausibility examination rather than a prediction accuracy study.

### 4.8. Diagrammatic Illustration

**Figure 4:**
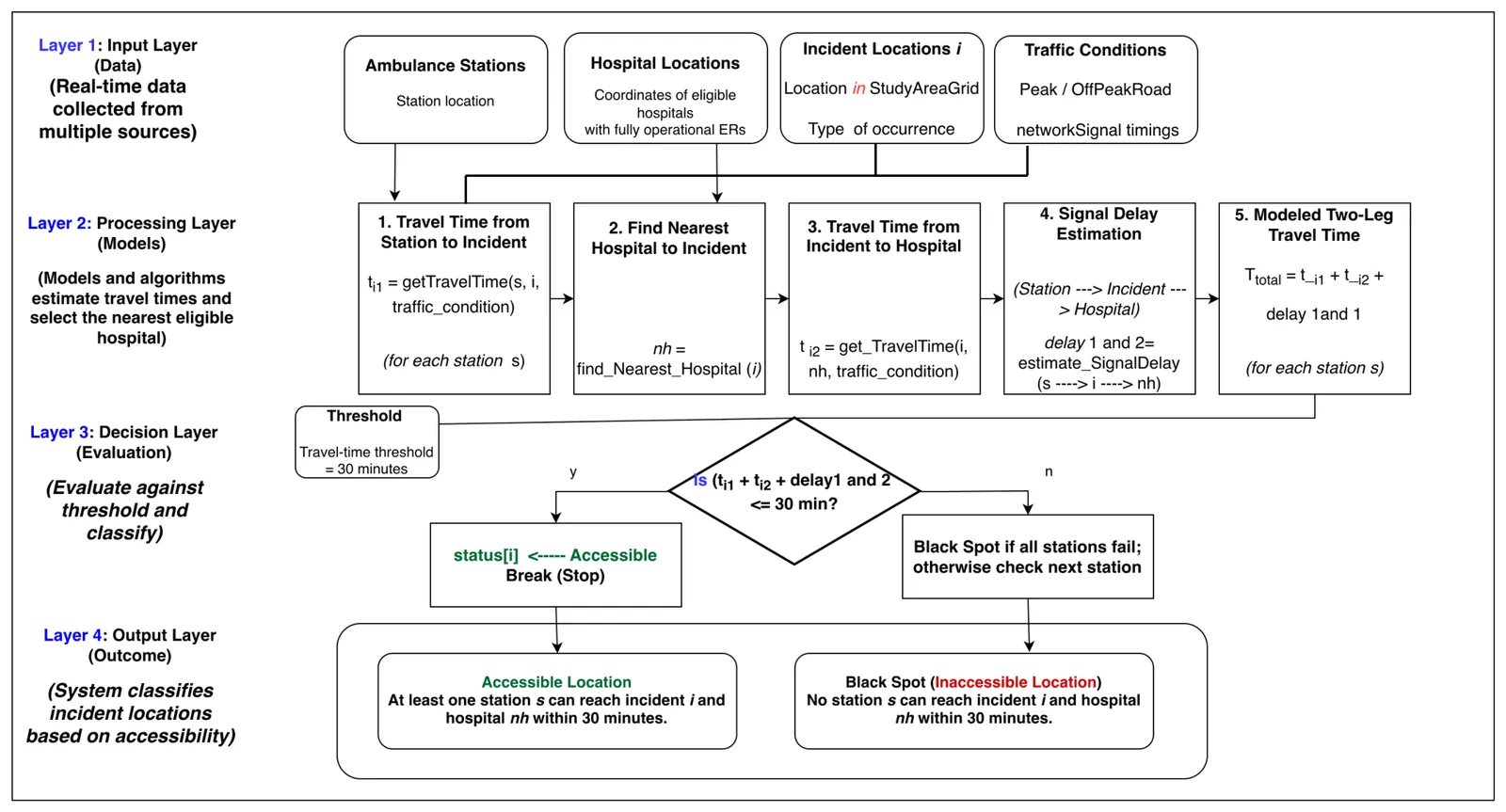
System model for evaluating emergency medical accessibility.

The system model in Figure 4 depicts the computational procedure used to evaluate the accessibility of emergency services throughout the study area. The process adopts a nested loop structure, iterating over different traffic conditions and geographic locations. The main steps of this procedure are:

- Start: initialize the model.
- Traffic condition loop: iterate over peak and non-peak hours.
- Ambulance-station loop: process each ambulance station in the study area.
- Incident location loop: loop through potential incident sites.
- Calculate travel time to incident: retrieve travel time from the ambulance station to the incident location using the Google Maps API.
- Find nearest hospital: identify the closest hospital with emergency services.
- Calculate travel time to hospital: retrieve the time required to travel from the incident location to the hospital.
- Estimate signal delay: calculate total expected red-light delays along the route.
- Calculate modeled two-leg travel time: sum all travel durations and delays.
- Decision: if this travel time is within 30 min, mark the location as accessible; otherwise, mark it as a black spot.
- Repeat the loops until all conditions and locations have been processed.
- End: for each scenario, generate output maps showing the accessible areas and black spots.

## 5. Result and Discussion

This section presents the empirical results of the analysis conducted to determine the accessibility of emergency healthcare under different traffic conditions. Travel-time-based isochrone modeling was applied for both peak and non-peak periods to capture the temporal variability of urban traffic and its impact on the efficiency of emergency responses. A comparison of the spatial coverage within a 30 min travel threshold during these time periods reveals how congestion influences hospital accessibility, identifies areas that remain consistently underserved, and highlights the zones whose accessibility deteriorates significantly during peak hours. The results are reported via spatial visualizations and statistical indicators, providing a comprehensive assessment of emergency service coverage and its sensitivity to traffic conditions.

**Figure 5:**
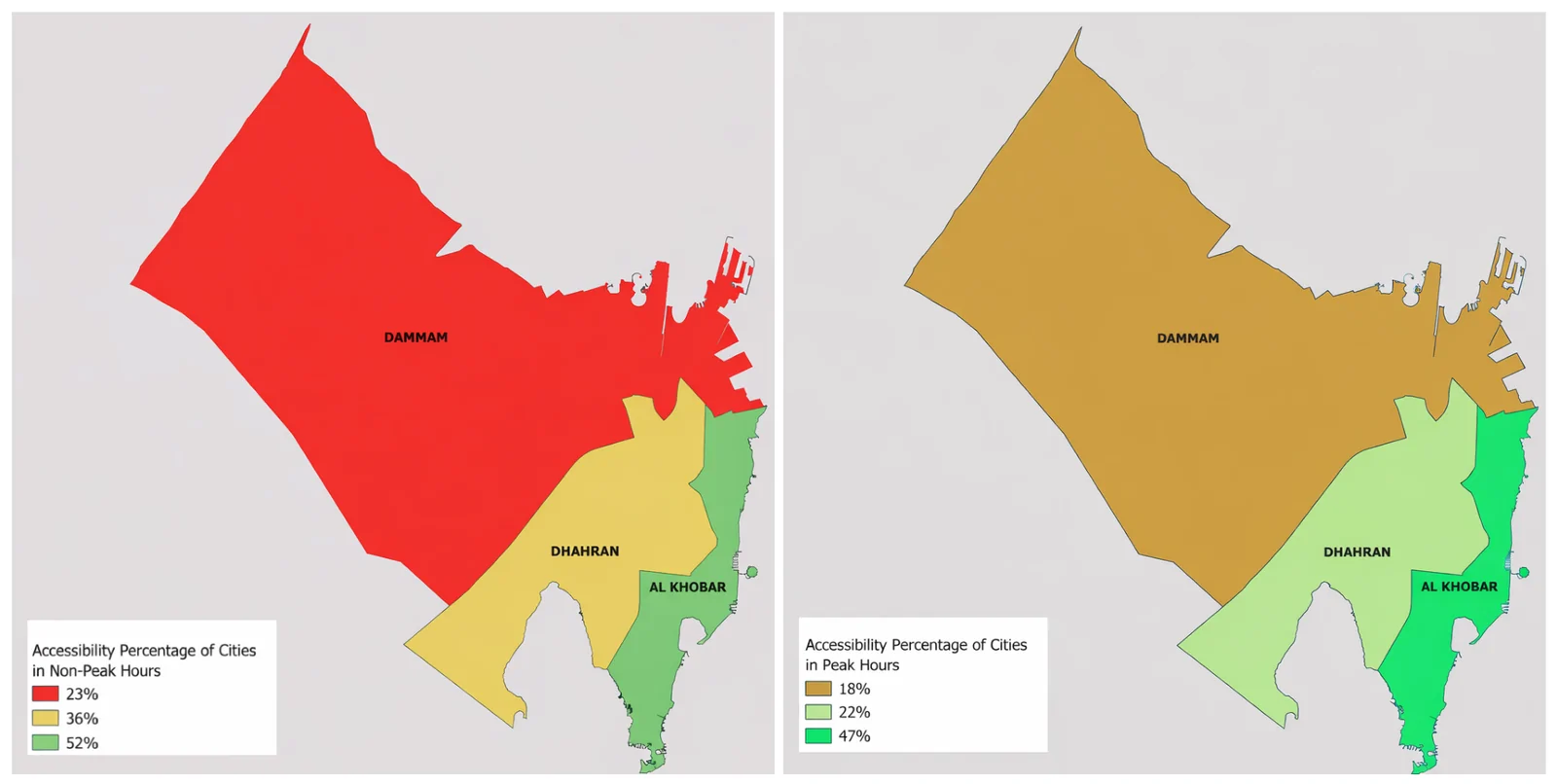
City-level geographic area coverage during non-peak and peak hours.

**Figure 6:**
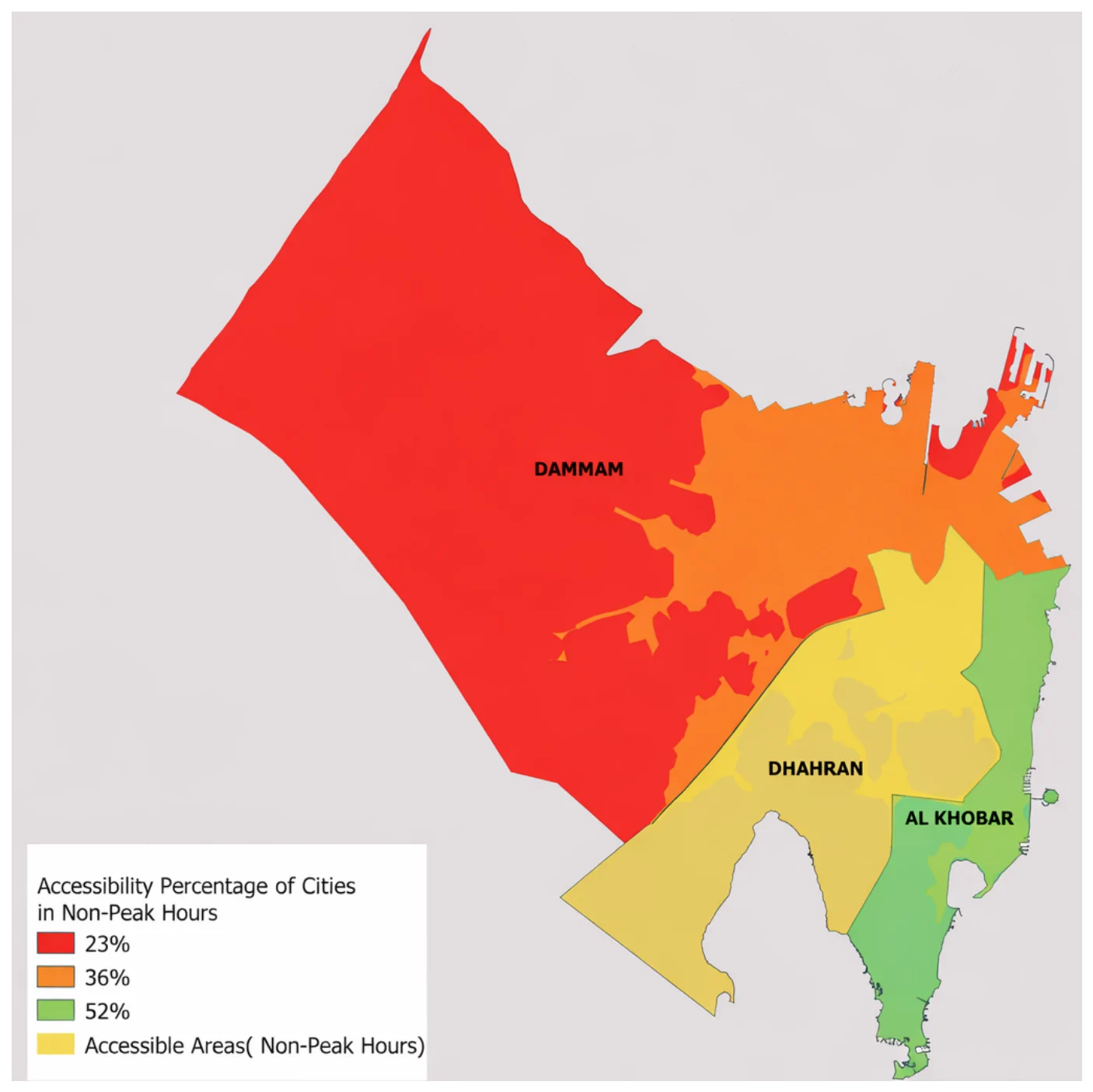
The non-peak hours in Dammam, Dhahran, and Al Khobar cities with accessibility areas shown.

The city-level percentages reference the percentage of the total study area of each city (using the modeled two-leg trip 30 min travel-time criterion) which is accessible, and does not indicate the population coverage. Figure 5 shows a significant disparity in accessibility between the three major cities, with Al Khobar consistently maintaining the highest levels of coverage. In non-peak hours, Al Khobar reaches an accessibility rate of 52%, which decreases slightly to 47% during peak hours. In stark contrast, Dammam has the lowest accessibility, decreasing from a mere 23% in non-peak hours to 18% during peak congestion. Dhahran occupies a middle ground, experiencing a notable drop from 36% during non-peak hours to 22% in peak periods. This data indicates that, although all cities suffer from traffic-induced delays, Dammam has a chronic lack of accessible coverage regardless of the time of day. Figure 6 shows the non-peak hours for each city together with accessibility areas. Notably, several neighborhoods depicted are emerging neighborhoods currently undergoing development, especially in Dammam city, which may be associated with their limited coverage.

**Figure 7:**
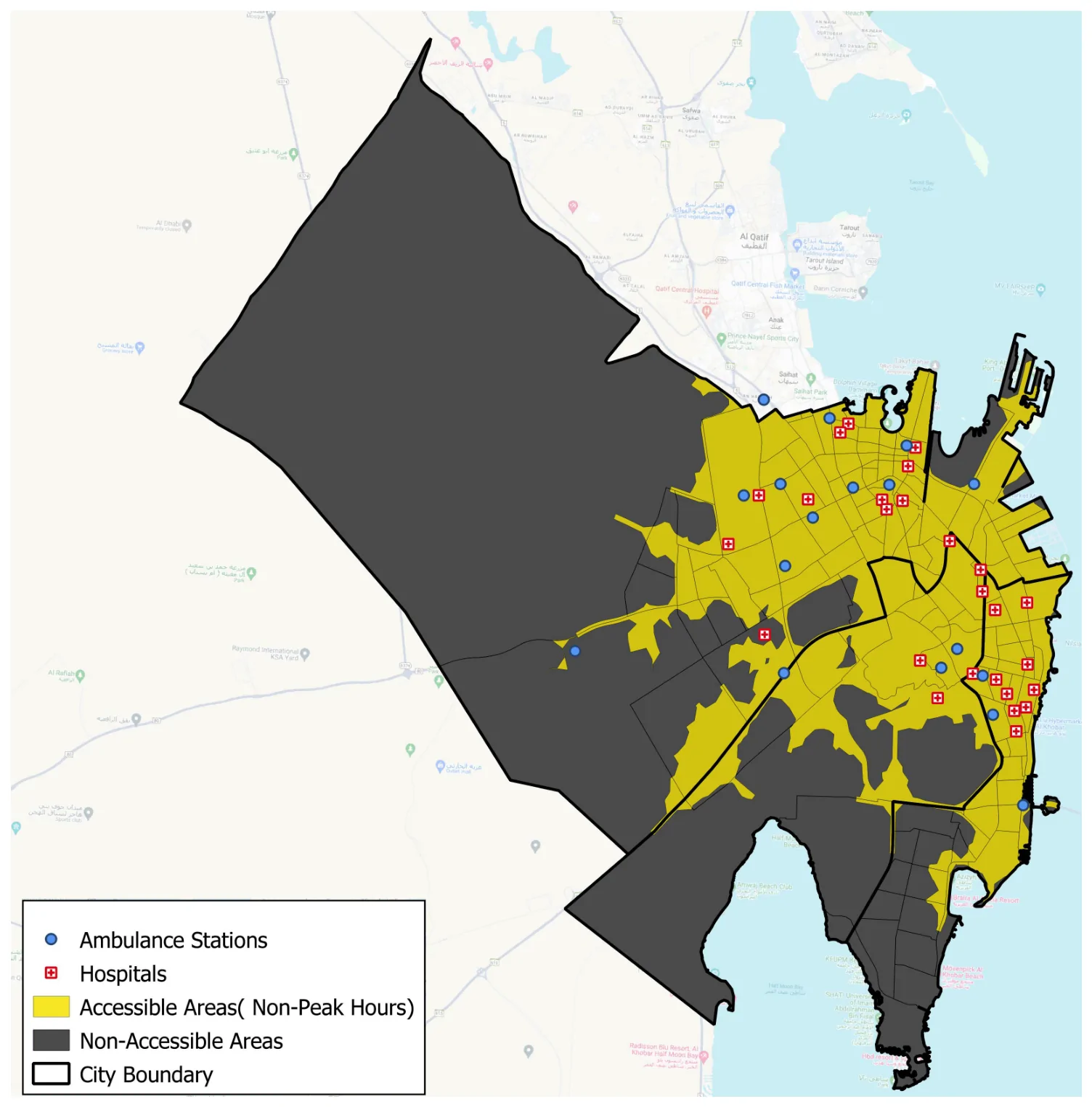
Emergency medical service accessibility during non-peak hours in Dammam, Dhahran, and Al Khobar.

The accessibility analysis indicates a marked difference in the emergency service coverage between periods of high and low traffic volumes. In the case of the latter (Figure 7), a considerable part of the city territory is located within the set response time criteria, with accessible areas (marked in yellow) covering much of the center and east regions. Generally, these areas correlate with the denser distribution of hospitals and ambulance stations, indicating that, under uncongested conditions, the current emergency infrastructure is usually able to provide timely responses to most of the more densely populated neighborhoods. Non-accessible areas (black spots) are mainly those in peripheral and less-developed zones, particularly toward the western and southern ends of the city, where sparse road connectivity and longer travel distances limit effective service reach. However, the black spot areas in Al Khobar are long-established neighborhoods. This suggests that accessibility issues in Al Khobar are not a product of recent urban expansion.

**Figure 8:**
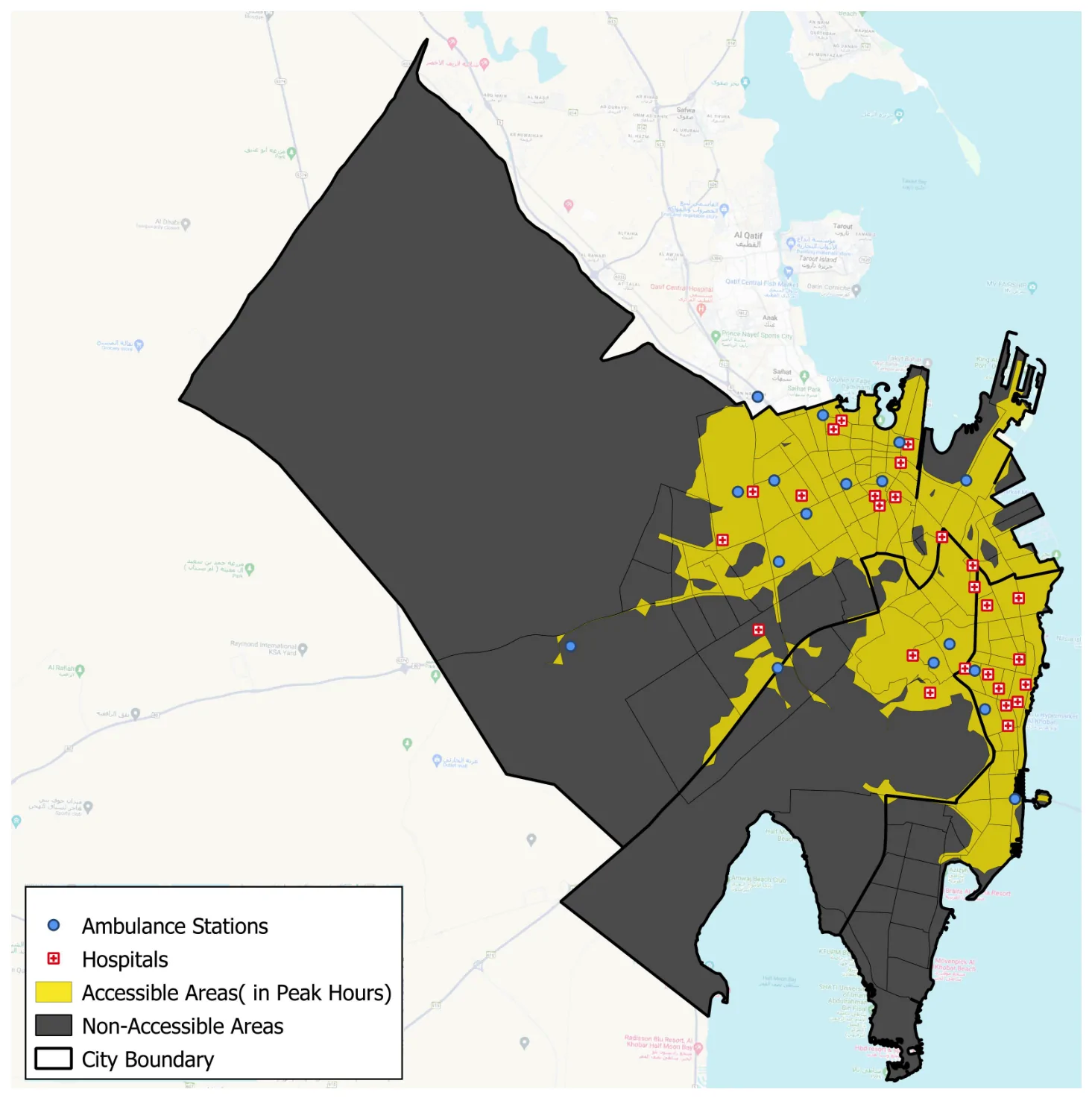
Emergency medical service accessibility during peak hours in Dammam, Dhahran, and Al Khobar.

Conversely, the peak-hour scenario (see Figure 8) demonstrates a pronounced decrease in the number of accessible areas, indicating the impact of traffic congestion on emergency response times. Accessibility gradually becomes segmented, with accessibility mostly occurring in regions near hospitals and along important roads. Some communities that are accessible in non-peak times turn out to be classified as black spots during peak hours, such as some parts of the inner city and transitional urban areas. This shift highlights the sensitivity of EMS performance to temporal traffic dynamics and demonstrates the vulnerability of certain districts during high-demand periods. Overall, the comparison results indicate that, although the baseline infrastructure may be adequate under favorable traffic conditions, peak-hour congestion significantly reduces equitable emergency access. This highlights the need for targeted interventions to improve resilience during peak demand, such as dynamic ambulance deployment, additional stations in underserved areas, or traffic-priority measures.

**Figure 9:**
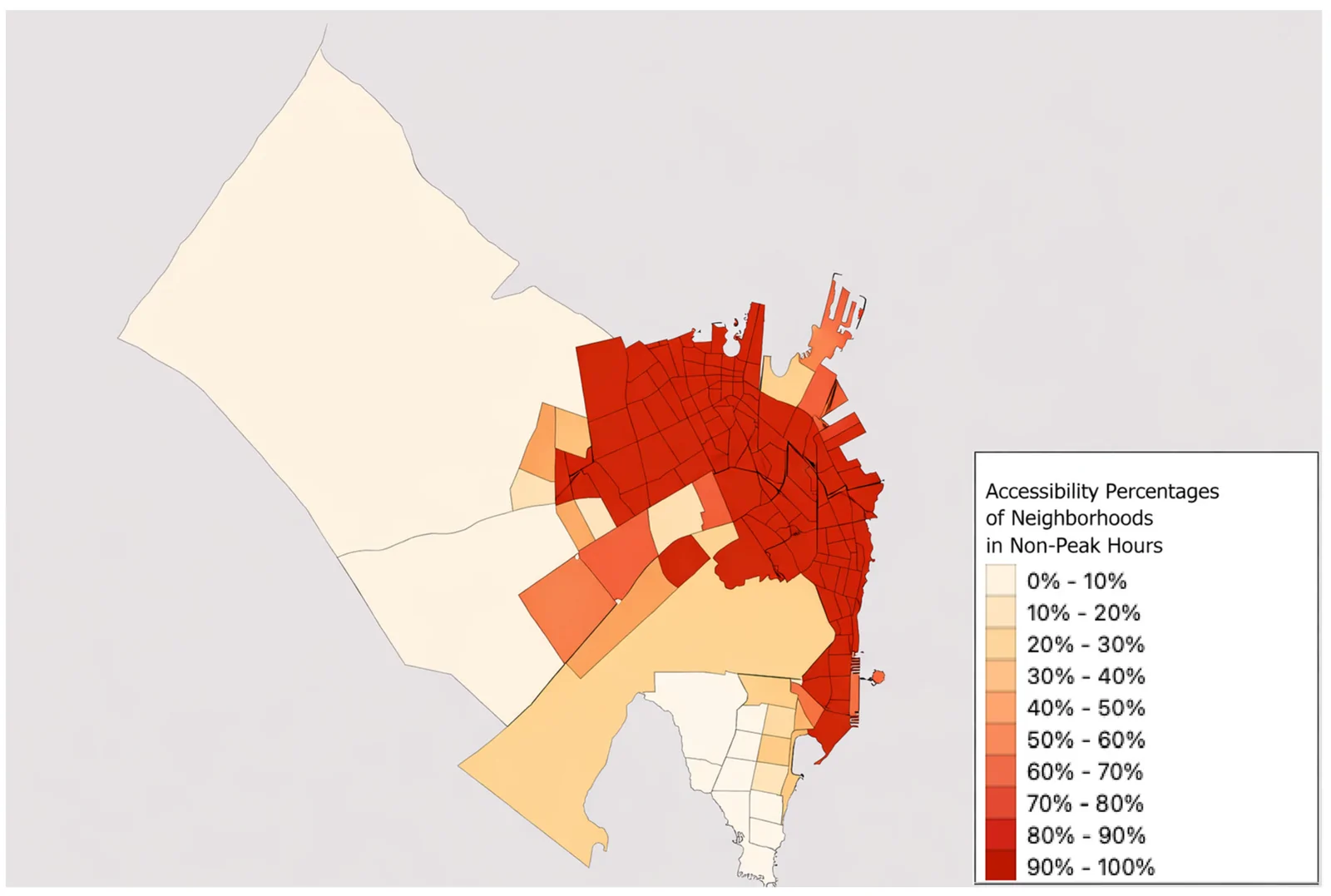
Neighborhood-level area accessibility during non-peak hours in Dammam, Dhahran, and Al Khobar.

**Figure 10:**
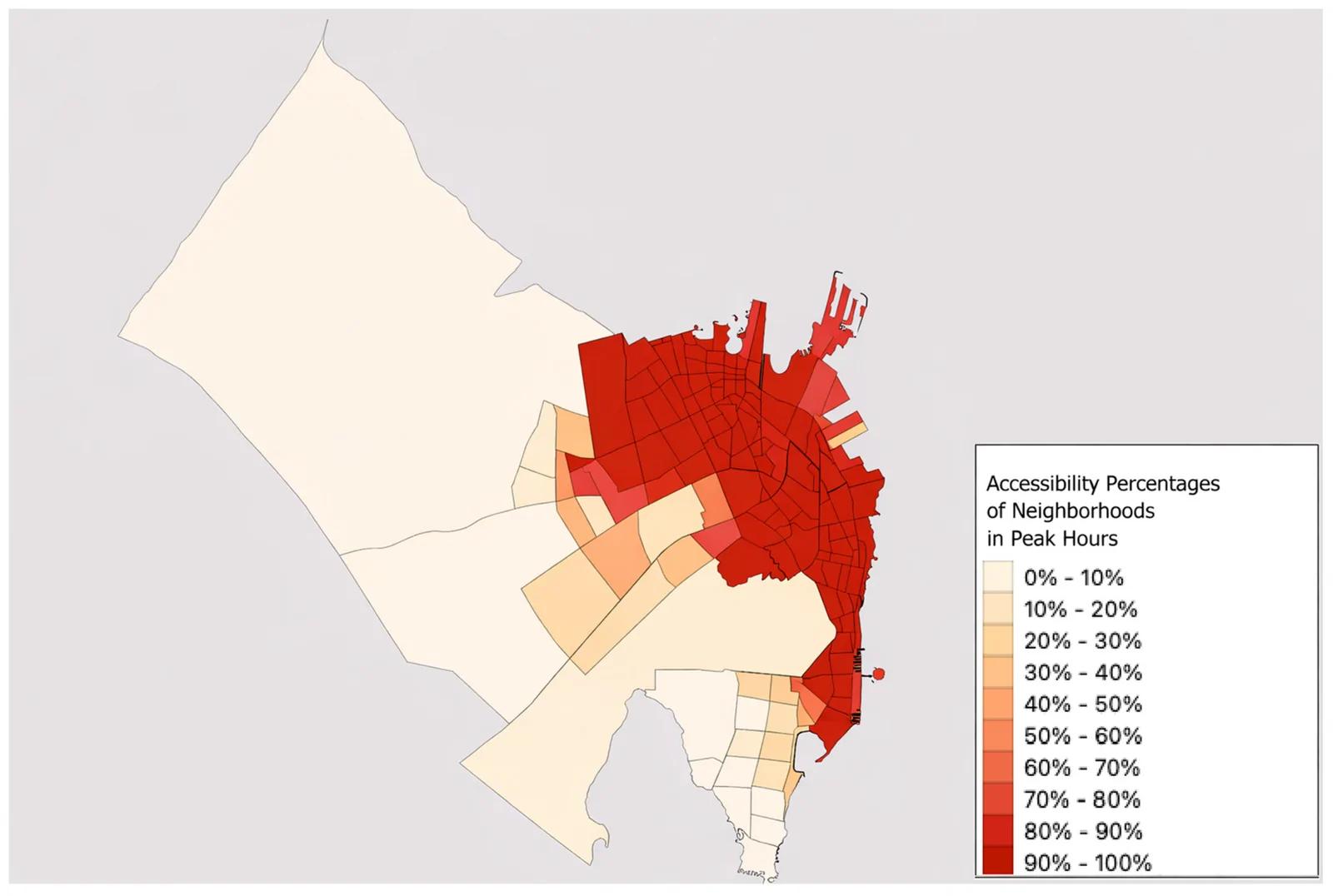
Neighborhood-level area accessibility during peak hours in Dammam, Dhahran, and Al Khobar.

The following figures illustrate accessibility on a per-neighborhood basis; each percentage indicates the accessible fraction of that neighborhood’s area, rather than the city as a whole. As shown in Figure 9 (non-peak hours) and Figure 10 (peak hours), the neighborhoods of Dammam, Dhahran, and Al Khobar have significantly different levels of accessibility to EMSs. The dense urban centers of Dammam and Al Khobar, where emergency facilities are closely integrated with a well-developed road network, have high levels (70–90%) of accessibility during non-peak hours. Conversely, several western and south-western neighborhoods in Dammam exhibit low accessibility despite their proximity to the urban center. These areas are either yet to be fully developed or have just become residential zones characterized by incomplete road infrastructure and limited connectivity, both of which may reduce the accessibility of services.

During peak-hour periods (Figure 10), most neighborhoods experience less accessibility, with a noticeable decrease in high-accessibility zones in central Dammam. Congestion significantly diminishes service coverage, even during non-peak times in well-served districts. Peripheral areas remain consistently underserved, although, in this regard, there is a clear distinction between cities. In southern Al Khobar, several fully developed residential neighborhoods still have low accessibility regardless of the time, indicating that their limited service reach may be associated with network structure, traffic congestion and location of facilities.

The contrasting accessibility patterns observed in Figure 9 and Figure 10 indicate the fundamentally different factors contributing to low accessibility within the metropolitan region. Accessibility problems in the western and southwestern districts of Dammam are mainly associated with inadequate urbanization. In particular, there are signs of continued growth of the residential sector in the western and southwestern parts of Dammam, coupled with insufficient integration of road connections, as well as inadequate access to arterial roads. Taken together, all these features impact the effectiveness of emergency services, even when it comes to non-peak hours.

On the other hand, the consistently poor accessibility seen in southern Al Khobar, even when fully residentially developed, suggests inherent constraints. These areas are well-integrated within an established urban matrix but continue to suffer from insufficient services, regardless of whether it is a peak period or not, suggesting that the impact of congestion, density of intersections, and improper location of facilities carry more weight than simply being developed. The difference underscores the need for different approaches, namely, infrastructure development and expansion of networks in the developing areas of Dammam, versus reducing congestion, prioritizing emergency travel, and relocation of facilities in the developed but poorly serviced areas of Al Khobar.

**Figure 11:**
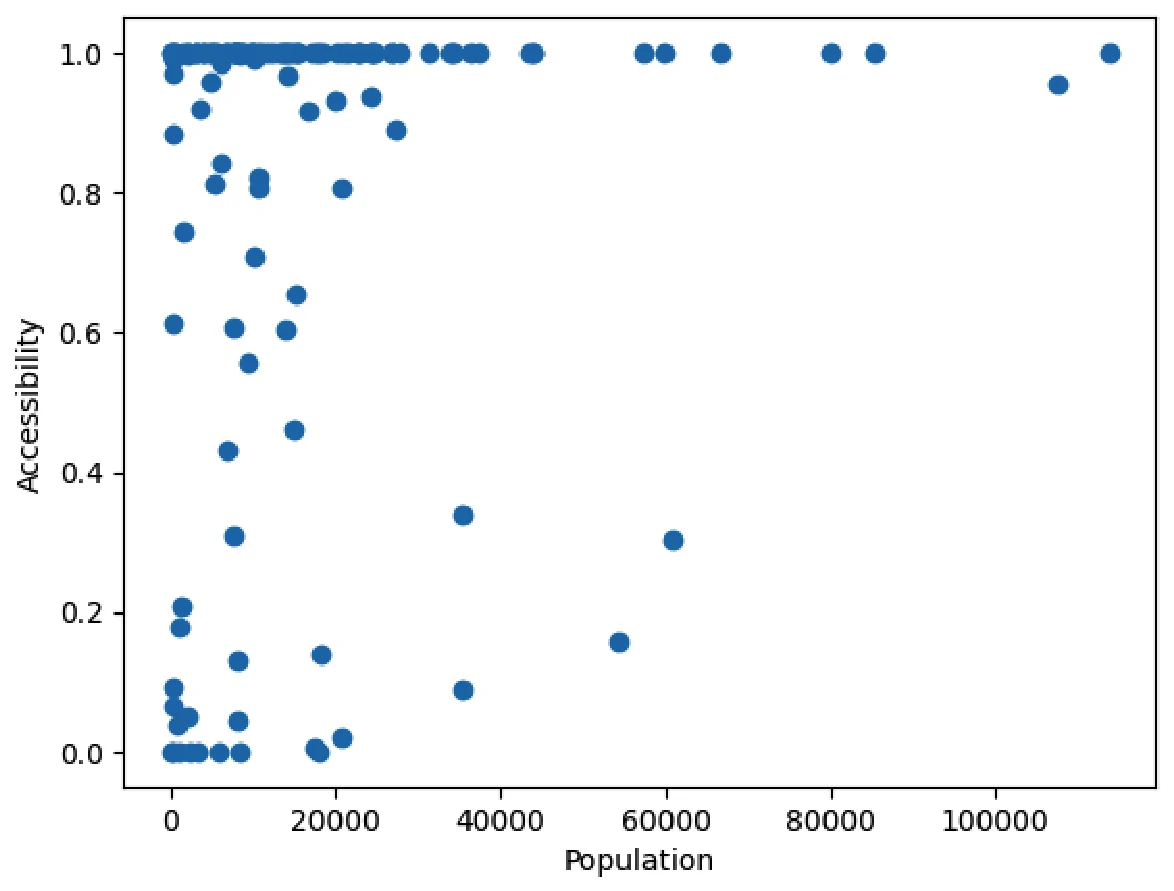
Neighborhood area accessibility versus population.

Figure 11 illustrates the relationship between accessibility and population in the various neighborhoods. According to the results, high levels of accessibility are not limited to low-population areas, as several densely populated neighborhoods have adequate accessibility. This indicates that there is sufficient emergency service coverage in key urban areas. However, the figure also shows neighborhoods that have moderate to high population sizes but relatively poor accessibility. These cases indicate a shortfall of localized services, leading to a considerable number of residents having less access to medical services. The absence of a clear linear relationship suggests that accessibility may not be directly determined by population size alone, but rather by a combination of factors, including road network structure, spatial distribution of services, and congestion.

**Figure 12:**
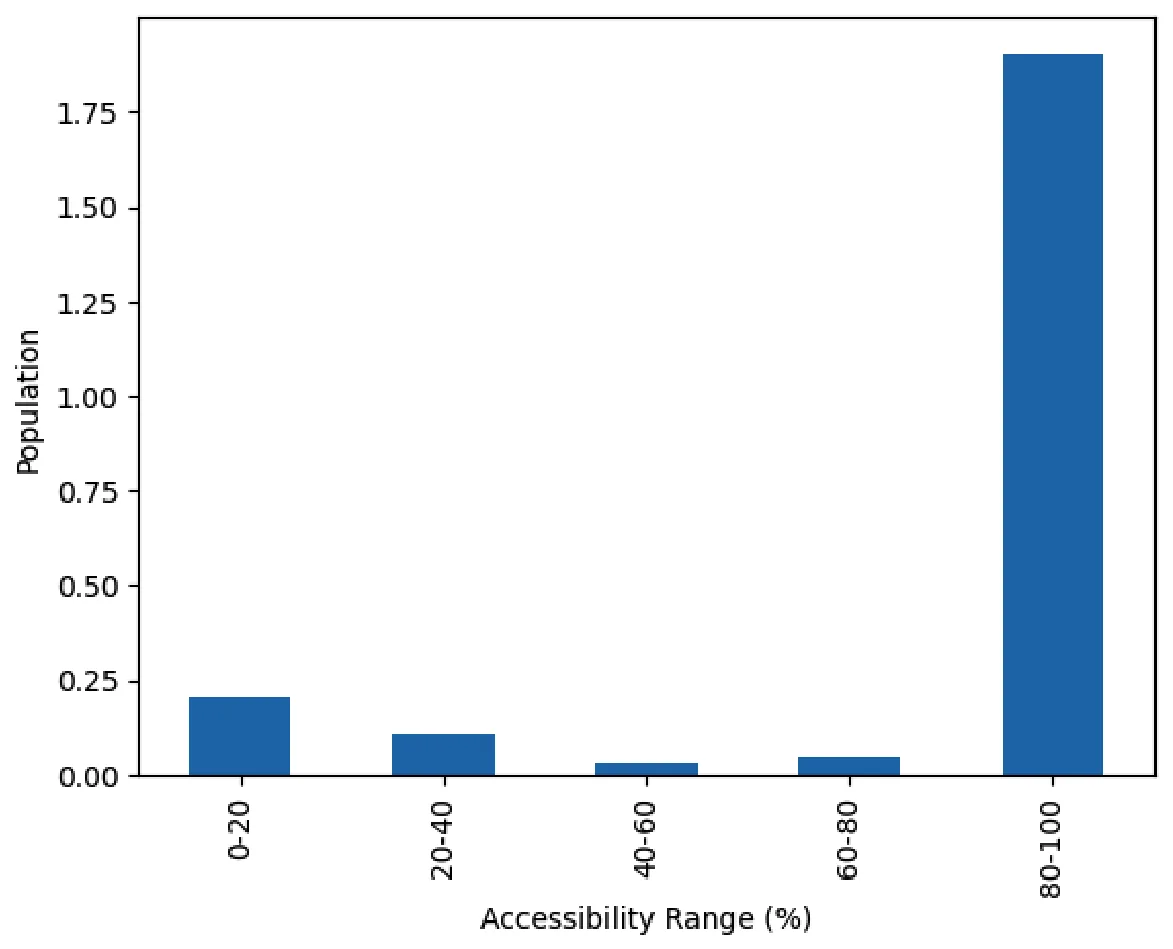
Distribution of neighborhood populations across predefined neighborhood area-accessibility ranges (0–20%, 20–40%, 40–60%, 60–80%, and 80–100%).

In order to demonstrate statistically the demographic impact of accessibility, neighborhoods were classified according to five accessibility thresholds. Within each category, the total exposed population was calculated with the following:

$$E_{k}=\sum_{j\in h}P_{j}$$

where $E_{k}$ represents the total population exposed to accessibility class *h*, and $P_{j}$ is the population of neighborhood *j* belonging to that class. The population of each neighborhood was allocated into the interval that had been determined based on its accessibility percentage value, and the totals thus obtained indicate the population of people living in neighborhoods categorized based on the accessibility instead of residents who have direct access. As shown in Figure 12, most of the population resides within the highest accessibility range (80–100%), indicating that a large proportion of residents live in neighborhoods with favorable accessibility conditions. However, a significant proportion of the population falls within lower accessibility ranges, particularly below 40%. This finding is important, as it demonstrates that such inequality is not due to spatial factors alone; it can also be attributed to demographics. When low-accessibility areas are geographically disadvantaged, a significant number of residents are affected, thereby increasing potential exposure to delayed emergency response.

**Figure 13:**
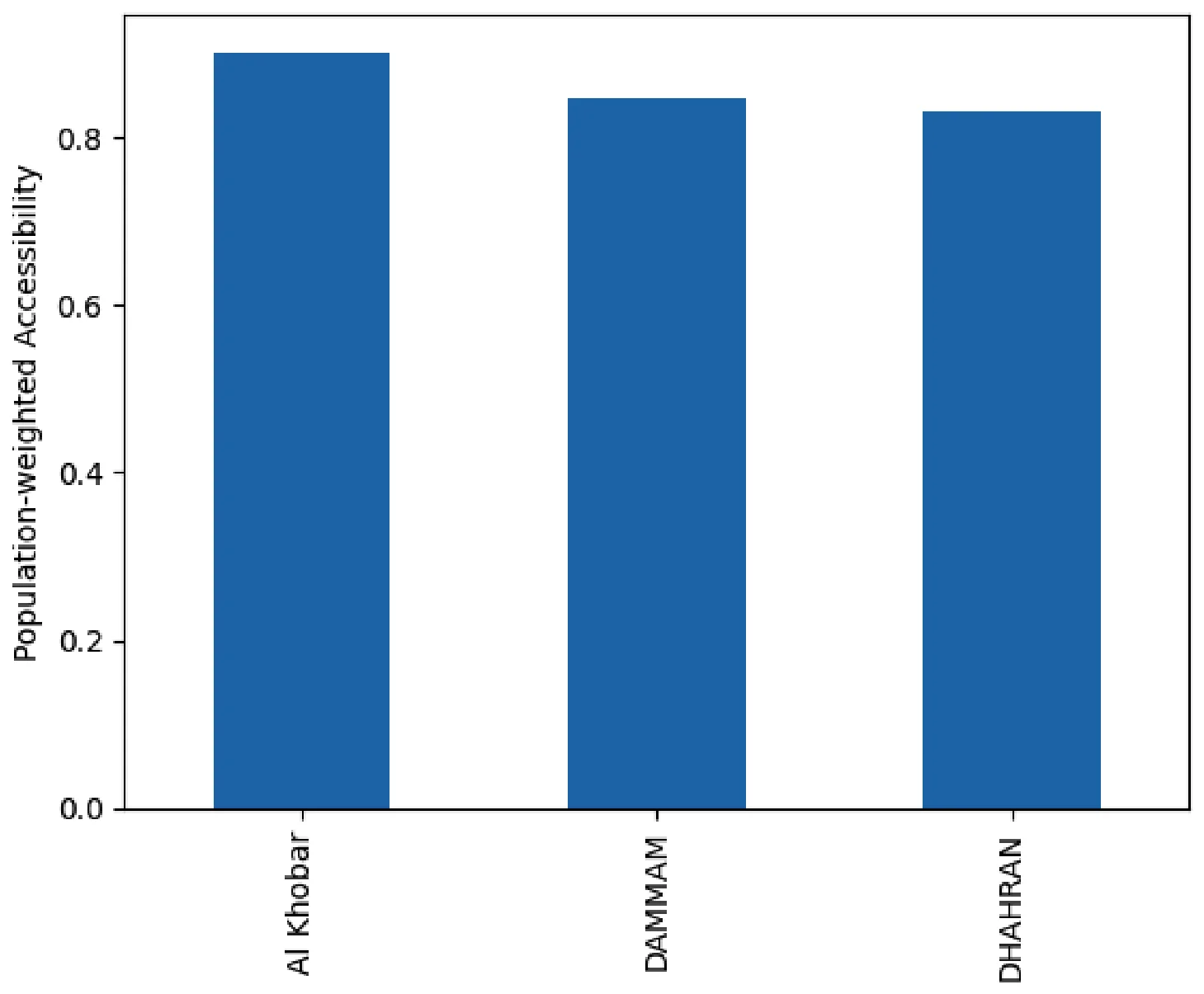
Population-weighted neighborhood area-accessibility index for Dammam, Al Khobar, and Dhahran.

From a population perspective, in order to determine accessibility, a population-weighted accessibility index was calculated using the following:

$$PW_{a}=\frac{\sum (A_{j}\cdot P_{j})}{\sum P_{j}}$$

where $A_{j}$ and $P_{j}$ represent the area-accessibility percentage and population of neighborhood *j*, respectively, with both sums taken over all neighborhoods in the corresponding city. Figure 13 shows that Al Khobar has the highest population-weighted accessibility, followed by Dammam and Dhahran. The moderate performance of Dhahran that has been observed elsewhere is with respect to geographic coverage; Dhahran has less population-weighted accessibility compared with Dammam. This determines that Al Khobar has higher population-weighted neighborhood area accessibility. The Al Khobar’s high accessibility can be attributed to its relatively compact urban structure and more efficient configuration of the road network. In contrast, slightly lower values in Dammam and Dhahran may be associated with the influence of urban expansion and infrastructure shortcomings.

**Figure 14:**
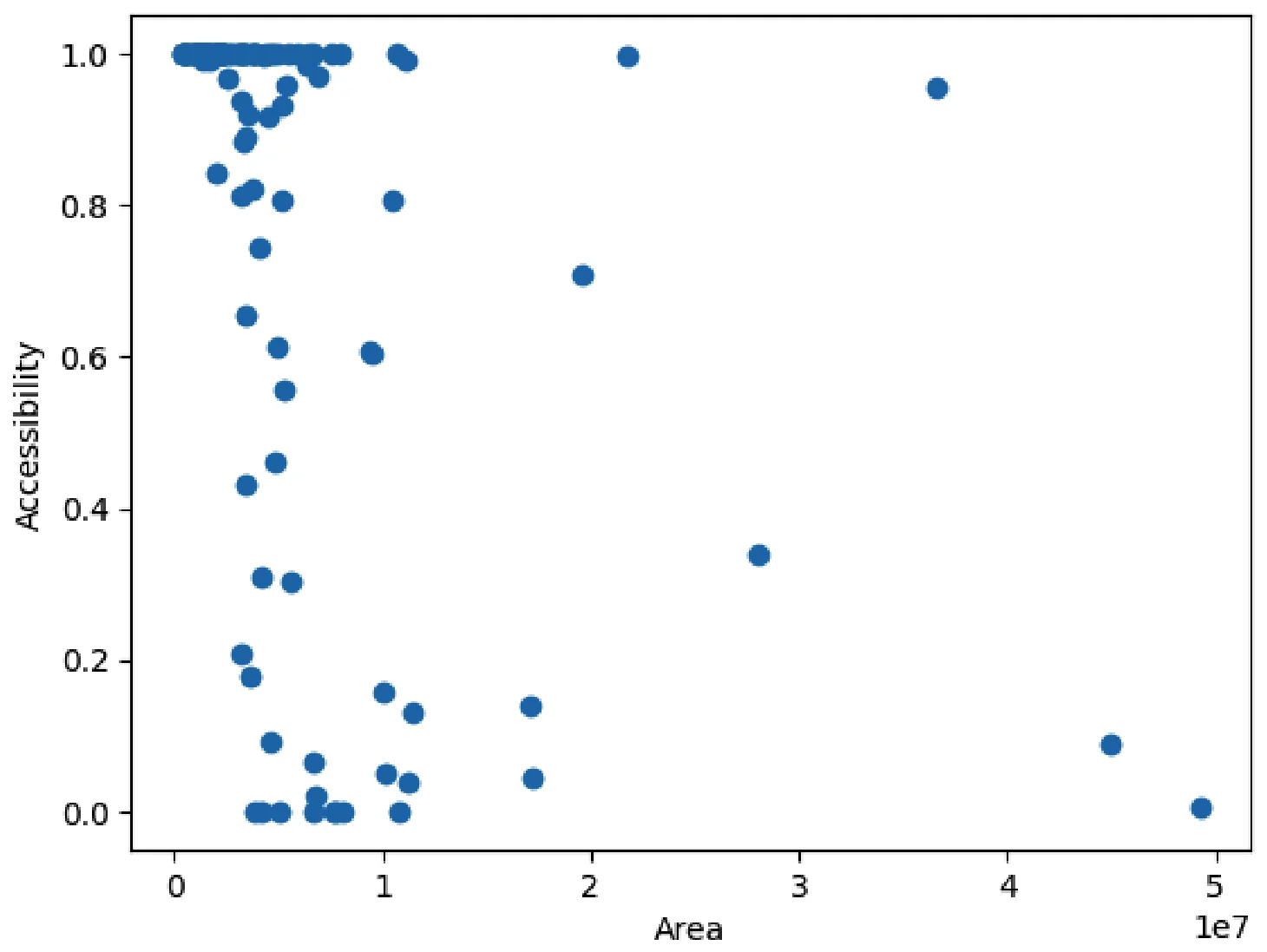
Relationship between neighborhood area size and accessibility levels.

Figure 14 shows the relationship between neighborhood size and accessibility. The results indicate that several large neighborhoods have low accessibility levels. This may be because these regions tend to be found in underdeveloped or semi-urbanized areas, with many being located in Dammam, where there is ongoing development in terms of infrastructure. On the other hand, smaller regions tend to have higher accessibility due to their proximity to the city center. Low accessibility might be associated with underdevelopment, and we cannot rule out the inefficiency in the transport system. These patterns imply that there may be a relationship between lower accessibility and some urban development. These explanations regarding urban development, road connections, general traffic, or facilities are interpretations of spatial patterns and not causality relationships tested separately. Note that the reported accessibility values are conditional on the model inputs and assumptions; there was no quantified validation through confidence intervals or error margins to values its uncertainty.

## 6. Conclusions and Future Work

This study presented a comprehensive spatial and population-based analysis of EMS accessibility in the cities of Dammam, Al Khobar, and Dhahran. By integrating GIS-based road network analysis with real-world travel-time data under both peak and non-peak conditions, the study determined whether modeled two-leg travel times were within 30 min. The results revealed significant spatial and temporal disparities in accessibility across the study area. While non-peak conditions had relatively wider coverage, peak-hour congestion reduced accessibility significantly, particularly in dense urban areas. The findings also highlighted notable differences between cities, with Al Khobar consistently having higher accessibility levels, while Dammam had persistent accessibility limitations regardless of traffic conditions. Dhahran showed intermediate geographic area coverage but was also sensitive to congestion impacts. The incorporation of population data also enabled the study to determine the demographic impact of accessibility. The population-weighted analysis demonstrated that, although a large number of residents live in neighborhoods with high area-accessibility levels, a considerable number of individuals are disadvantaged by factors that produce low-access conditions. Importantly, the results identified two possible types of accessibility challenges: (i) the development-related limitations in expanding and partially developed areas, particularly in Dammam, and (ii) the structural and operational constraints in fully developed and densely populated neighborhoods, especially in Al Khobar. Overall, the study emphasizes that accessibility inequalities may be associated with both spatial and demographic factors, and that an assessment of EMS travel accessibility requires that both transportation dynamics and population distribution be considered. The proposed framework provides a practical and scalable method for identifying underserved areas and supporting evidence-based decision-making when planning urban expansion and transportation.

The additional signal-delay term was not adjusted using signal-level measurements and considered as a limitation of the model and it may partially double count delays already reflected in the map traffic estimates. Some possible directions for future research might include the following: since independent quantitative validation remains a limitation of the study, future work will be an important direction in which to work on quantitative aspects, including correlation coefficient, confidence interval and others. Furthermore, the inclusion of real-time traffic and dynamic routing information may improve the time-based aspect of the accessibility measurement. Also, the model can be expanded with regard to other factors, such as the number of ambulances available, response times, and hospitals’ capacity (bed availability), thus allowing one to conduct a more thorough analysis of the effectiveness of the emergency services. Further research could explore alternative approaches to optimization in locating new ambulance stations and hospitals. Additional ambulance stations, relocation, and traffic-priority measures need to be examined through modeled cases before implementation; the current analysis study determines the accessibility gaps (we called them black spots) but does not indicate the efficiency of these interventions.

## Data Availability

Data supporting this study are available from the corresponding author upon request, subject to third-party permissions and data-use restrictions. Aggregate accessibility results are reported in the manuscript.
